# Introduction to the *RSC Advances* themed collection on nano and microscale modifications of biomaterials

**DOI:** 10.1039/d4ra90035a

**Published:** 2024-04-10

**Authors:** Andrzej Zieliński, Beata Majkowska-Marzec

**Affiliations:** a Gdańsk University of Technology, Institute of Manufacturing and Materials Technology, Department of Biomaterials Technology Poland andrzej.zielinski@pg.edu.pl

## Abstract

Andrzej Zieliński and Beata Majkowska-Marzec introduce the *RSC Advances* themed collection on nano and microscale modifications of biomaterials.
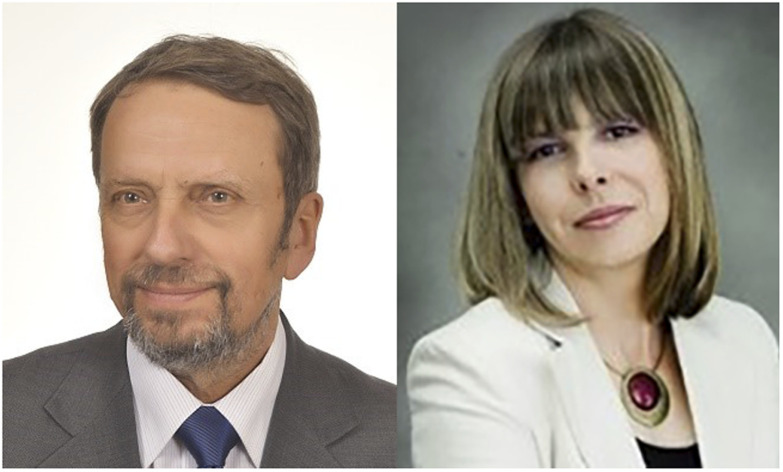

The dramatically increasing development of novel biomaterials, their manufacturing techniques, and surface modifications, as well as their application in *in vivo* tests and clinical trials, is evident. It is interesting to note in what a relatively short time the well-known long-term implants have become medical standard: titanium dental implants started in 1965, and total hip arthroplasty (not very successful) was first attempted in 1961. Even after about fifty years of research, real progress can be noticed recently thanks to composite materials and coatings, the development of their fabrication such as 3D printing, and, last but not least, the appearance of sophisticated instruments to characterize surfaces, *e.g.* atomic force microscopy. For implants and materials for bone repair, the role of an interface between any materials is key. The bulk properties are important to assess the durability of the implant as a whole construct, and the interface dictates the corrosion rate, hydrophilicity and biocompatibility, bioactivity, antibacterial properties, and the lack of cytotoxicity. All these surface properties determine the function and lifetime of long-term implants. And, all of them are modeled at the nano and microscale.

This themed collection published in *RSC Advances* presents the most recent achievements in nano and microscale modifications of the surfaces of biomaterials. The materials and coatings represent all groups such as metallic, ceramic, polymer, and especially composites. The most important features include microstructure and topography, nano and micro dimensions, and chemical and phase compositions of the surface layers and coatings. Therefore, research progress in this specific field can be highly diverse, as demonstrated by the most recent reviews which focus on the nanocomposites of gelatin and various nanoparticles,^1^ nano-based biomaterials for leiomyosarcoma therapy,^2^ dynamic biomaterials based on material nanoarchitectonics,^3^ hydroxyapatite nanoparticles and nanostructured materials,^4^ intelligent biomaterials for micro and nanoscale additive manufacturing,^5^ nanoscale surface coatings and topographies for neural interfaces,^6^ and polymeric drug delivery systems including hydrogels, nanofibers, and microspheres.^7,8^

In this themed collection, made up of review articles and research papers, the emphasis is firmly placed on the demonstration of links between nano and microscale surface modifications, and their effects on mechanical, physical, chemical, and biological properties. The reviews focus on general presentations of surface modifications of implants, nanoparticles used in wound healing, ceria-based coatings for biodegradable magnesium alloy, and hydrogels used for bone repair. The research papers highlight advances in areas such as polymer-ceramic 3D printed composites, freeze-drying produced silane-polymer composites, curcumin-oxide nanoparticles on graphitic-carbon nitrides, cellulose-based electrodes with manganese oxide, carbon nanotubes, and titania composite coatings, and a 3D-printed cardiac spheroidal droplet-based system used to assess the maximum toxicity level of chemotherapeutic drugs.

The collection of published papers includes an excellent review of the application of surface-modified biomaterials proposed by Hu and co-workers. The most important task is to reduce the risk of early failures of implants and increase their lifetime, mainly by prevention of infections. The described surface modifications include the deposition of thin layers and thicker coatings, covalent grafting, the appearance of self-assembled monolayers, plasma surface modification, and other strategies. The review discusses the effects of surface modification techniques on different properties of biomaterials, in particular on cytocompatibility, antibacterial efficiency, antifouling, and hydrophobicity (https://doi.org/10.1039/d3ra02248j).

In wound healing studies, novel therapeutic approaches have been proposed to deliver effective treatment. Nanoparticle-based materials are preferred due to their antibacterial activity, biocompatibility, and increased mechanical strength in wound healing. They can be divided into six main groups, each showing several advantages and disadvantages. Better wound care/healing techniques are now possible, thanks to the development of wound healing strategies based on these materials, which mimic the extracellular matrix microenvironment of the wound. All these questions are discussed in the review made by Dam and co-workers, especially when NPs have been used in wound healing and how this strategy has become a key biotechnological procedure to treat skin infections and wounds (https://doi.org/10.1039/d3ra03477a).

Another interesting study described by Padmanabhan and co-workers focuses on the effect of curcumin-loaded ZnO nanoparticles settled on graphitic-carbon nitride sheets against breast cancer. The hexagonal wurtzite ZnO phase nanoparticles were distributed on base nanosheets with good mutual adhesion of both components. The obtained composite showed good thermal stability. The release of curcumin from the composite was faster at acidic pH compared to neutral. The biomaterial demonstrated antibacterial activity against *E. coli* and *S. aureus*, and anticancer activity. The lactate dehydrogenase activity assay disclosed the cytotoxicity of the nanocomposite (https://doi.org/10.1039/d3ra02887a).

We both express our deep gratitude to all the authors for their amazing contributions. We hope that all these papers will be very interesting to many researchers who work on the development of biomaterials based on the application of nano and microscale modifications to their further improvement.

## Supplementary Material
